# Downregulation of Iron–Sulfur Cluster Biogenesis May Contribute to Hyperglycemia-Mediated Diabetic Peripheral Neuropathy in Murine Models

**DOI:** 10.3390/antiox13091036

**Published:** 2024-08-26

**Authors:** Lin Wu, Fei Huang, Zichen Sun, Jinghua Zhang, Siyu Xia, Hongting Zhao, Yutong Liu, Lu Yang, Yibing Ding, Dezhi Bian, Kuanyu Li, Yu Sun

**Affiliations:** 1 Jiangsu Key Laboratory of Molecular Medicine, Medical School, Nanjing University, Nanjing 210093, China; 2 Endocrinology Department, Yancheng First People’s Hospital, Affiliated Hospital of Medical School, Nanjing University, Yancheng 224000, China; 3 State Key Laboratory of Pharmaceutical Biotechnology, Department of Vascular Surgery, Nanjing Drum Tower Affiliated Hospital of Medical School, Nanjing University, Nanjing 210008, China; 4 Suqian Scientific Research Institute of Nanjing University Medical School, Nanjing University, Suqian 223800, China

**Keywords:** iron–sulfur cluster biogenesis, frataxin, IscU, DPN, inflammation

## Abstract

Background: Diabetic peripheral neuropathy (DPN) is considered one of the most common chronic complications of diabetes. Impairment of mitochondrial function is regarded as one of the causes. Iron–sulfur clusters are essential cofactors for numerous iron–sulfur (Fe-S)-containing proteins/enzymes, including mitochondrial electron transport chain complex I, II, and III and aconitase. Methods: To determine the impact of hyperglycemia on peripheral nerves, we used Schwann-like RSC96 cells and classical *db/db* mice to detect the expression of Fe-S-related proteins, mitochondrially enzymatic activities, and iron metabolism. Subsequently, we treated high-glucose-induced RSC96 cells and *db/db* mice with pioglitazone (PGZ), respectively, to evaluate the effects on Fe-S cluster biogenesis, mitochondrial function, and animal behavior. Results: We found that the core components of Fe-S biogenesis machinery, such as frataxin (Fxn) and scaffold protein IscU, significantly decreased in high-glucose-induced RSC96 cells and *db/db* mice, accompanied by compromised mitochondrial Fe-S-containing enzymatic activities, such as complex I and II and aconitase. Consequently, oxidative stress and inflammation increased. PGZ not only has antidiabetic effects but also increases the expression of Fxn and IscU to enhance mitochondrial function in RSC96 cells and *db/db* mice. Meanwhile, PGZ significantly alleviated sciatic nerve injury and improved peripheral neuronal behavior, accompanied by suppressed oxidative stress and inflammation in the sciatic nerve of the *db/db* mice. Conclusions: Iron–sulfur cluster deficiency may contribute to hyperglycemia-mediated DPN.

## 1. Introduction

Diabetic peripheral neuropathy (DPN), one of the most common chronic complications of diabetes, impacts approximately half of individuals with type 1 diabetes (T1D) and type 2 diabetes (T2D) [1]. Furthermore, DPN has been linked to significant morbidity, such as insomnia, anxiety, depression, and an increased risk of foot or ankle fractures, ulceration, and lower-limb amputations [2]. A distal symmetrical type of DPN affects the great majority of its sufferers. Long-term hyperglycemia and metabolic abnormalities, including mitochondrial dysfunction, oxidative stress, neuroinflammation, accumulation of advanced glycation end products, and dyslipidemia (an increase in low-density lipoprotein (LDL) and triglycerides and a decrease in high-density lipoprotein (HDL)) are the main risk factors for the development of DPN [2]. Schwann cells are sheath cells of peripheral nerve fibers, which wrap around the axons of peripheral nerve fibers and form myelin sheaths. They are crucial in maintaining the function of peripheral nerve cell axons, and the maintenance of this function vitally depends on mitochondria producing enough ATP and metabolites [3].

Mitochondrial iron–sulfur (Fe-S) cluster biogenesis is a mitochondrial essential and minimal function to provide Fe-S clusters to numerous essential mitochondrial, cytosolic, and nuclear Fe-S proteins, explaining the essentiality of mitochondria [4,5]. Fe-S clusters act as essential cofactors in mitochondrial complexes I, II, and III for the functionality of the electron transport chain (ETC) and in aconitase for the tricarboxylic acid (TCA) cycle. Loss or deficit of the Fe-S cluster compromises mitochondrial function. ISCU, a scaffold protein, and frataxin (FXN), a nucleus-encoded mitochondrial protein, are the two core components of Fe-S cluster biogenesis machinery. ISCU mutation-mediated mis-splicing dominantly in muscle causes myopathy with exercise intolerance [6]. It has been demonstrated in a study that ISCU depletion leads to inadequate Fe-S and mitochondrial dysfunction. FXN deficiency in humans causes Friedreich ataxia (FRDA), an inherited degenerative syndrome characterized by central and peripheral neuropathy, accompanying cardiomyopathy, with a high incidence of diabetes [4,7]. In vitro studies indicate that FXN-dependent accumulation of unspecified reactive oxygen species [8] depends on the concurrent reduction in antioxidants in frataxin-deficient states [9]. Postural imbalance and ataxia result from degenerating the dentate nucleus in the cerebellum, dorsal root ganglia (DRG), and peripheral nerves [10,11]. Therefore, Fe-S cluster deficiency might play a critical role in DPN occurrence.

Pioglitazone (PGZ), a member of the thiazolidinedione (TZD) family, is an insulin-sensitizing agent that has been reported to have antidiabetic effects [12]. Diabetic mouse studies have shown that TZD treatment improves diabetic and neurological outcomes and decreases the infarct area, irrespective of the timing of therapy or the ischemic method used [13]. Clinical data show that PGZ administration is associated with a significant reduction in the risk of diabetes and dementia [14]. The long-term benefits of PGZ also include improved renal outcomes in type 2 diabetes [15].

In this study, high glucose was unexpectedly found to significantly downregulate Fxn and IscU expression in vitro and in vivo. Here, we test a hypothesis that hyperglycemia-mediated downregulation of Fe-S cluster biogenesis contributes to DPN and that PGZ provides neuroprotective effects by upregulating Fe-S cluster biogenesis in *db/db* mice, a suitable model for obese humans with T2D complicated by progressive neuropathy.

## 2. Materials and Methods

### 2.1. Animals

Male C57BLKS (WT) and *db/db* mice were obtained from the Model Animal Research Center of Nanjing University (Nanjing, China) and fed standard chow for 16 weeks to establish the DPN model. For PGZ treatment, the mice aged 12 weeks were treated with vehicle (dimethyl sulfoxide, DMSO) or PGZ (25 mg/kg/d, oral administration by gavage) for 4 weeks. The animals were housed in groups under standard conditions with a 12 h light–dark cycle and a temperature of 25 °C. All experiments involving animals were subject to review and approval by the Animal Investigation Ethics Committee of Nanjing University and were conducted per the Guidelines for the Care and Use of Laboratory Animals published by the National Institutes of Health, USA.

After anesthesia, the leg muscles of the mice were stripped, and the sciatic nerves (SCNs) were collected. One-half of the SCN was placed in formaldehyde solution for tissue histology and another half was stored in an −80 °C freezer for subsequent testing.

### 2.2. Glucose and Insulin Tolerance Tests

The mice underwent a 12 h fasting period and were injected intraperitoneally with 1.5 g/kg of glucose. Blood samples were collected from the tail vein and determined at 0, 15, 30, 60, 90, and 120 min during the glucose tolerance test (GTT). The mice were fasted for 6 h and intraperitoneally injected with insulin (0.75 U/kg body weight) (Sigma, St. Louis, MO, USA) for the insulin tolerance test (ITT). Glucose levels were examined at 0, 15, 30, 60, and 90 min after injection.

### 2.3. Hot Plate Test

A hot plate test was performed as previously described [16]. In brief, each mouse was acclimated before being placed on a hot plate set at 50 °C, 52 °C, or 56 °C. The time it took for the mouse to either lick its hind paw or jump was noted. Each measurement was taken at least four times, with a minimum 20 min gap between each.

### 2.4. Rotarod Test

Balance and coordination motor functions were evaluated by utilizing an accelerating rotarod (Jiangsu SANS Technology Co., Ltd., Nanjing, China) as described in a previous study [17]. In short, the mice were positioned on the rod, and the rotation began at 4 revolutions per minute for 30 s and then it was gradually increased at a rate of 10 revolutions per minute. A 10 min break was given to each mouse between each test. All mice underwent training three times a day prior to the test. The time it took for the mice to fall after acceleration was measured.

### 2.5. Beam-Walk Test

A beam-walk test was performed as previously described [18]. The coordination capabilities of the mice were assessed through the beam-walk test, which involved using 22 mm diameter horizontal wooden beams. The mice underwent 3 training sessions, and the time at which they passed the 1 m long wooden beam was measured. The test was repeated four times, with a minimum of 5 min between each repetition.

### 2.6. Electron Microscopy

The animals were euthanized following drug treatment and then perfused with 0.9% phosphate-buffered saline (PBS) transcardially. The sciatic nerves were subsequently fixed in 2% Paraformaldehyde in 0.1 M cacodylate at room temperature for 30 min and then stored at 4 °C. Before further processing, it is essential to rinse the cells once or twice. The samples underwent dehydration using a series of ethanol concentrations ranging from 50% to 100%, followed by replacement with propylene oxide. Subsequently, the samples were immersed in a mixture of 50% propylene oxide and 50% Epon resin for 1 h and then in pure Epon. The following day, the previous step was repeated with fresh Epon. The samples were then placed in molds or Beem capsules with fresh Epon, ensuring the removal of air bubbles, and left in the oven at 60 °C for a minimum of 24 h. Finally, the samples were examined using a JEM 1230 (JEOL Ltd., Tokyo, Japan) transmission electron microscope at 80 kV, and images were captured using a Gatan Multiscan 791 (Gatan Inc., Pleasanton, CA, USA) digital camera.

### 2.7. Histological Assays

The tissues were fixed in 4% paraformaldehyde and sectioned into 2 to 7 μm thick samples. Hematoxylin–eosin (H&E) staining was performed as previously reported [17].

### 2.8. Ferrozine Iron Assays

Iron contents in RSC96 cells and sciatic nerve tissue were detected via the ferrozine iron assay as previously described [19]. In short, 22 μL concentrated HCl (11.6 M) was combined with 100 μL of homogenized tissue samples containing approximately 500 μg total protein. The mixture was heated to 95 °C for 20 min and then followed by centrifugation at 12,000× *g* for 10 min. The resulting supernatant was carefully transferred to a new tube. Ascorbate was introduced to convert Fe^3+^ to Fe^2+^. After 2 min incubation at 25 °C, ferrozine and saturated ammonium acetate (NH_4_Ac) were successively added to each tube, and the absorbance was measured at 570 nm (BioTek ELx800, Shanghai, China) within a 30 min timeframe.

### 2.9. Labile Iron Pool (LIP) Measurements

Labile iron was quantified using iron probes calcein-AM (Aladdin, Shanghai, China) or rhodamine B-[(1,10-phenanthroline-5-yl)-aminocarbonyl]benzyl ester (RPA, Squarix GmbH, Marl, Germany) as previously reported [20]. To measure the cytosolic LIP, 100 µM 2,2′-bipyridyl (BIP), an iron chelator, was introduced to extinguish the calcein–iron complex. The rise in fluorescence following the addition of BIP was denoted as the level of LIP in the cytoplasm. As for the mitochondrial LIP, the quenching of RPA by iron was observed after adding the specific iron chelator pyridoxal isonicotinoyl hydrazone (PIH, final concentration 2 mM) for 30 min. The disparity in the fluorescence before and after PIH chelation represents the mitochondrial LIP.

### 2.10. The Enzymatic Activities of Aconitase, Complexes I and II, and Mitochondrial Membrane Potential (MMP) Levels

The aconitase activity was determined using a method described in a previous study [17]. The activities of complexes I and II were evaluated following the manufacturer’s instructions. Complex I was obtained from Abcam, while Complex II was purchased from Comin Biotechnology Co. (Suzhou, Jiangsu, China). MMP level was assessed using a JC-1 mitochondrial membrane potential detection kit (Solarbio Science & Technology Co., Ltd., Beijing, China).

### 2.11. ROS Detection Assays

To assess the production of reactive oxygen species (ROS) in RSC96 cells, an ROS assay kit (YEASEN, Shanghai, China) was used to test ROS production. DCFH-DA was diluted with serum-free cell culture medium at a ratio of 1:1000 to achieve a final concentration of 10 μM. Then, the cells collected via centrifugation were mixed with a 1 mL diluted probe. After incubation at 37 °C for 30 min in the dark, the mixture was centrifuged at 3000 rpm for 5 min, and 200 μL of supernatant was added into 96-well plates. Subsequently, the fluorescent signal of each sample was measured at 485 nm excitation/530 nm emission using the microplate reader. Three technical repeats were conducted for each concentration.

### 2.12. Western Blot Assays

The mouse tissues underwent lysis using RIPA lysis buffer and were homogenized using a KZ-II 2100 rpm High-Speed Tissue Homogenizer. They were obtained from Wuhan Servicebio Technology Co., Ltd., Wuhan, China. The protein concentration was determined with Bradford buffer. Total proteins were loaded (20–40 µg/lane), separated on SDS-PAGE gels at 100 V, transferred to nitrocellulose membranes, and then incubated with primary and secondary antibodies for analysis. Detection of proteins was performed using Tanon Science and Technology Co., Ltd. (Shanghai, China) ECL-plus reagent.

The information for primary antibodies is as follows: anti-NFS1 (Proteintech, Inc., Chicago, IL, USA, cat# 15370-1-AP), anti-Ndufs1 (Proteintech, Inc., Chicago, IL, USA, cat# 12444-1-AP), anti-SDHB (Abcam, Cambridge, MA, USA cat# 178423), anti-UQCRFS1 (Proteintech, Inc., Chicago, IL, USA, catalog number 18443–1-AP), anti-Rabbit Aco2 (Proteintech, Inc., Chicago, IL, USA, cat# 11134–1-AP), anti-TfR1 (Zymed, San Francisco, CA, USA, cat# 136800), anti-Fpn1 (Alpha Diagnostic, San Antonio, TX, USA, cat# MTP11-A), anti-Ftl (Abcam, Cambridge, MA, USA cat# 69090), anti-Nrf2 (Cell Signaling Technology, Shanghai, China, cat# 12721S), anti-Ho-1 (Cell Signaling Technology, Shanghai, China, cat# 70081S), anti-catalase (Cell Signaling technology, Shanghai, China, cat# 12980), anti-β-Actin (Bioworld, Louis Park, MN, USA cat# AP0060), and anti-Fxn, IscU, Irp1 and Fth (polyclonal, self-made, raised from rabbits). All self-made antibodies were validated in previous studies [19,20].

### 2.13. Quantitative Real-Time PCR

The tissues or cells were processed to extract total RNA with the RNA isolator Total RNA Extraction Reagent (Vazyme Biotech, Nanjing, China) and then converted to cDNA using HiScript^®^ III RT SuperMix for qPCR (Vazyme Biotech, Nanjing, China), following the provided manufacturer’s protocols. Subsequent RT-qPCR assays were carried out utilizing ChamQ Universal SYBR qPCR Master Mix (Vazyme Biotech, Nanjing, China) in an Applied Biosystems ViiA™7 system.

The following primers were used: forward primer 5′-TGAACAACGATGATGCACTTG-3′ and reverse primer 5′-CTGAAGGACTCTGGCTTTGTC-3′ for mouse Il-6, forward primer 5′-ACGTCGTAGCAAACCACCAA-3′ and reverse primer 5′-GCAGCCTTGTCCCTTGAAGA-3′ for mouse TNF-α, forward primer 5′-CAGGCAGGCAGTATCACTCA-3′ and reverse primer 5′-AGGCCACAGGTATTTTGTCG-3′ for mouse Il-1β, and forward primer 5′-GGCTACCACATCCAAGGAA-3′ and reverse primer 5′-GCTGGAATTACCGCGGCT-3′ for mouse 18S as a control.

### 2.14. Statistics Analysis

The data were expressed as the mean ± SD. Each experiment was conducted independently more than three times. Statistical analysis was carried out using GraphPad Prism 8, utilizing either Student’s *t*-test or one-way analysis of variance (ANOVA). Significance was considered at *p* < 0.05.

## 3. Results

### 3.1. High Glucose Downregulates Fe-S-Cluster Biogenesis and Fe-S-Containing Enzymatic Activities in RSC96 Cells

**Figure 1:**
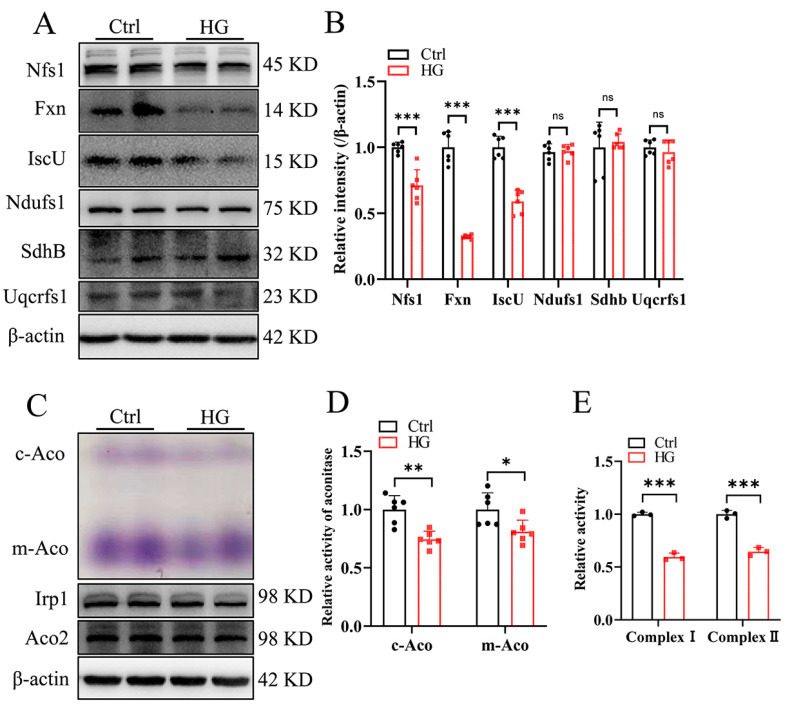
High-glucose treatment downregulates iron–sulfur (Fe-S) cluster biogenesis and activities of Fe-S-containing enzymes in RSC96 cells. (**A**–**D**), RSC96 cells were cultured in DMEM with 5.5 mM glucose (Crtl) or 50 mM glucose (high glucose, HG) for 2 d. (**A**,**B**) The expression of the iron–sulfur cluster-related proteins detected via Western blotting (**A**), quantified in (**B**). (**C**,**D**) Cytosolic aconitase (c-Aco) and mitochondrial aconitase (m-Aco) activities (purple density) and the corresponding protein levels (pair set: c-Aco and Irp1; m-Aco and Aco2), quantified in (**D**). (**E**) The activities of mitochondrial complexes I and II. Data were normalized to the Ctrl group. Values are shown as the mean ± SD. n = 3–6. The *t*-test was used for significance. KD: molecular weight in kilodaltons, the same in later figures. ^ns^ *p* > 0.05, * *p* < 0.05, ** *p* < 0.01, and *** *p* < 0.001.

RSC96 cells, spontaneously transformed rat Schwann-like cells, derived from a long-term culture of rat primary Schwann cells, were used to establish DPN Schwann cell injury in vitro through culturing in a medium with high glucose (HG, 50 mM) [21]. After HG treatment, we detected the protein levels of a few core components of Fe-S cluster biogenesis machinery. Among them, Nfs1 (cysteine desulfurase), Fxn, and IscU showed a significant decrease to around 75%, 30%, and 55%, respectively (Figure 1A, quantified in Figure 1B). Consistent with this, HG treatment reduced the activities of both cytosolic and mitochondrial aconitases (Figure 1C, quantified in Figure 1D), which rely on the availability of Fe-S clusters. Mitochondrial complexes I and II of the respiratory chain are the most abundant Fe-S cluster-containing enzymes in mitochondria. Similarly, HG treatment reduced complex I and II activities (Figure 1E). These results suggested that HG treatment suppresses Fe-S cluster biogenesis to inhibit mitochondrial function.

### 3.2. High-Glucose Treatment Increases the Cellular Labile Iron Pool and Promotes ROS Production in RSC96 Cells

**Figure 2:**
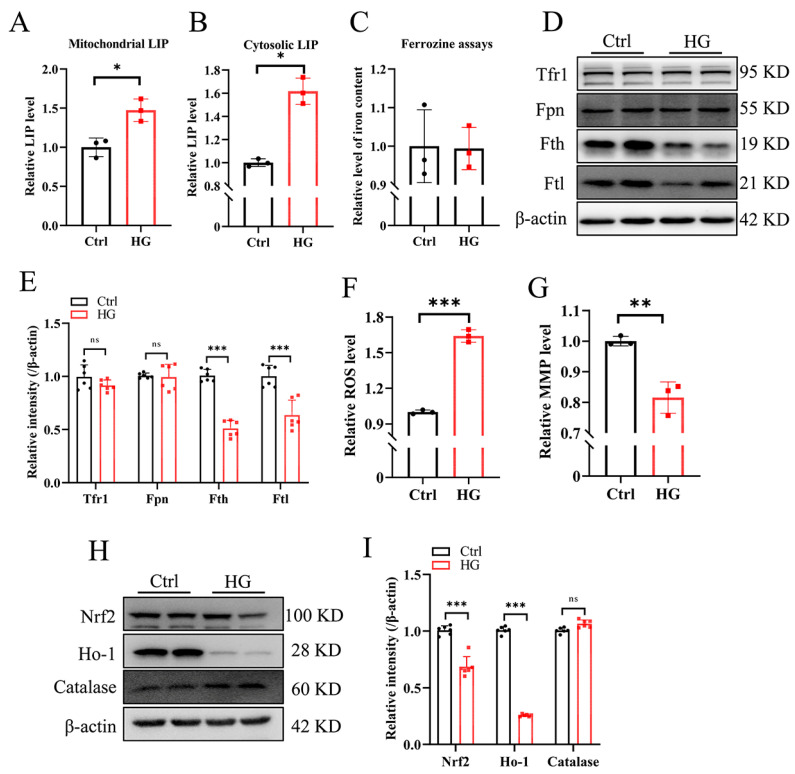
High-glucose treatment increases the cellular labile iron pool and promotes ROS production in RSC96 cells. (**A**–**G**), RSC96 cells were cultured in DMEM with 5.5 mM glucose (Crtl) or 50 mM glucose (HG) for 2 d. (**A**) The relative levels of cytosolic LIP (labile iron pool) measured using calcein-AM along with 2,2′-bipyridyl (BIP). (**B**) The relative levels of mitochondrial LIP measured with RPA along with pyridoxal isonicotinoyl hydrazine (PIH). (**C**) Cellular iron content detected via the ferrozine iron assays. (**D**,**E**) The protein expression of Tfr1, Fpn, Fth, and Ftl detected via Western blotting, quantified in (**E**). (**F**) The relative levels of reactive oxygen species (ROS). (**G**) The relative mitochondrial membrane potential (MMP). (**H**,**I**) The expression levels of oxidative stress-related proteins Nrf2, Ho-1, and catalase detected via Western blotting, quantified in (**I**). Data were normalized to the Ctrl group. Values are shown as the mean ± SD. n = 3–6. The *t*-test was used for significance. ^ns^ *p* > 0.05, * *p* < 0.05, ** *p* < 0.01, and *** *p* < 0.001.

We wonder whether the reduction in Fe-S biogenesis is attributed to iron deficiency. Then, the intracellular iron and oxidative stress were evaluated. Surprisingly, the labile iron pool (LIP) was increased in both the cytosol and mitochondria (Figure 2A,B). However, the cellular iron did not alter between the control (Ctrl) and HG group based on the ferrozine assays (Figure 2C), which revealed the labile iron and ferritin iron. Interestingly, the expression levels of ferritin subunits H and L (Fth and Ftl) significantly decreased around half after HG treatment, whereas Trf1 and Fpn remained constant (Figure 2D, quantified in Figure 2E), suggesting an increased Fenton reaction rate to produce ROS, a crucial pathophysiological pathway of DPN [22]. After HG treatment, the ROS levels significantly increased by ~60%, as detected by a fluorescent DCFH-DA probe (Figure 2F), and the mitochondrial membrane potential (MMP) decreased by ~20% (Figure 2G). Then, the expression of oxidative stress-related proteins was detected, and we found a significant decrease in heme oxygenase 1 (Ho-1) and nuclear factor erythroid 2-related factor 2 (Nrf2), but not catalase (Figure 2H, quantified in Figure 2I). These results indicate that the elevated production of ROS was caused by the increase in labile iron after HG treatment.

### 3.3. Pioglitazone Treatment Increases the Expression of Genes Involved in the Biosynthesis of Fe-S Clusters and Recovers Iron Homeostasis and Redox Balance in RSC96 Cells

**Figure 3:**
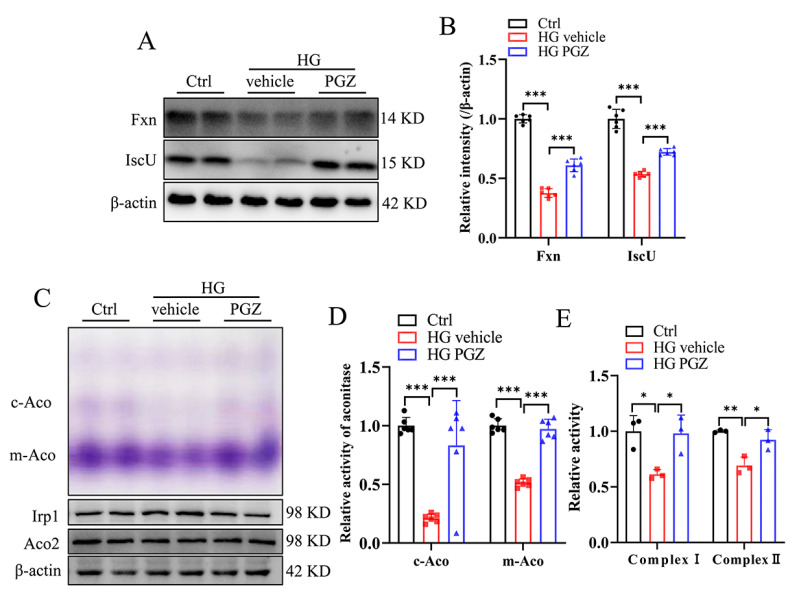
Pioglitazone treatment increases the expression of genes involved in the biosynthesis of iron–sulfur clusters in RSC96 cells. RSC96 cells were subjected to high glucose (50 mM, HG) for 2 d with or without pioglitazone (10 μM, PGZ). (**A**,**B**) The protein expression levels of Fxn and IscU detected via Western blotting (**A**), quantified in (**B**). (**C**) Cytosolic aconitase and mitochondrial aconitase activities (purple density) and the corresponding protein levels, quantified in (**D**). (**E**) The activities of mitochondrial complexes I and II. Data were normalized to the Ctrl group. Values are shown as the mean ± SD. n = 3–6. ANOVA was used for significance. * *p* < 0.05, ** *p* < 0.01, and *** *p* < 0.001.

Multiple PPARγ agonists, including PGZ, are capable of increasing the expression of FXN in the models of Friedreich Ataxia [23,24], a neurological disorder caused by FXN deficiency. To investigate whether PGZ treatment affects the expression of FXN in the DPN cell model, we treated the RSC96 cells with high-concentration glucose (HG, 50 mM) for 24 h with or without PGZ (10 μM) and found that PGZ treatment increased the protein levels of Fxn and IscU in HG-treated RSC96 cells (Figure 3A, quantified in Figure 3B). Consistent with this, the enzymatic activities of both cytosolic and mitochondrial aconitases and mitochondrial complexes (I and II) were significantly higher after PGZ treatment (Figure 3C–E). These results demonstrate that PGZ restored the synthesis of Fe-S clusters and enzymatic activities of Fe-S proteins.

**Figure 4:**
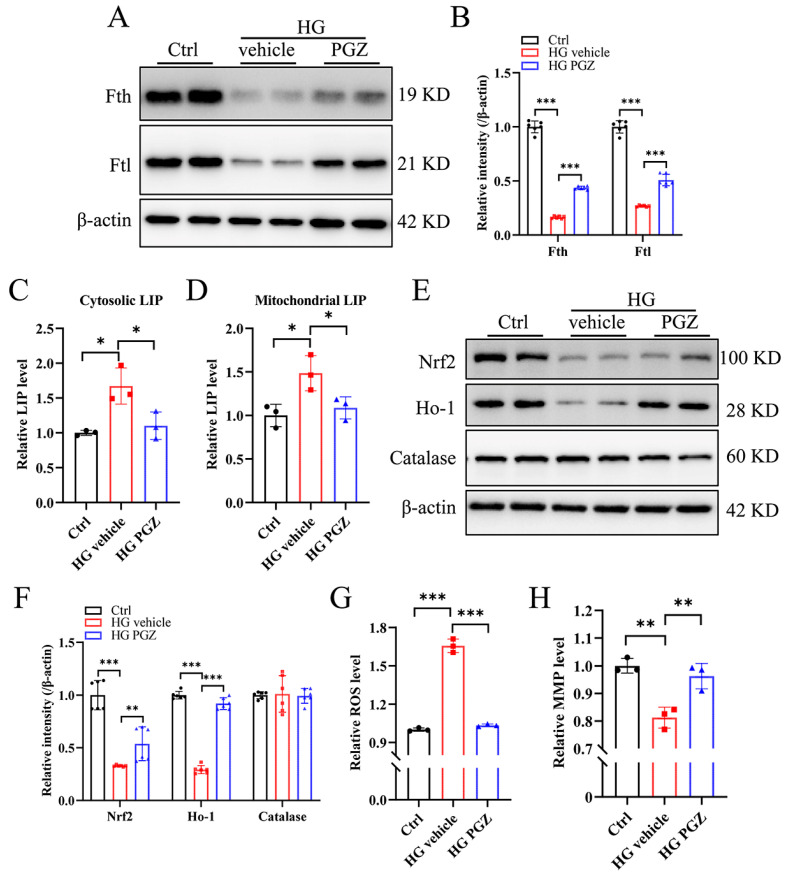
Rescue of Fe-S cluster biogenesis through pioglitazone treatment recovers iron homeostasis and redox balance in RSC96 cells. (**A**) The protein expression of Fth and Ftl after pioglitazone (PGZ) treatment, quantified in (**B**). (**C**,**D**) The relative levels of cytosolic and mitochondrial LIP, respectively. (**E**) The expression levels of oxidative stress-related proteins Nrf2, Ho-1, and catalase detected via Western blotting, quantified in (**F**). (**G**) The relative cellular levels of ROS. (**H**) The relative MMP. Data were normalized to the Ctrl group. Values are shown as the mean ± SD. n = 3–6. ANOVA was used for significance. * *p* < 0.05, ** *p* < 0.01, and *** *p* < 0.001.

The stability of the Fe-S cluster is sensitive to intracellular levels of ROS. The restoration of Fe-S enzymatic activities by PGZ suggests the recovery of cellular LIP and oxidative stress. As shown in Figure 4A–D, PGZ treatment significantly increased the protein expression of Fth and Ftl and decreased the cytosolic and mitochondrial LIP. In addition, the expressions of Nrf2 and Ho-1 increased after PGZ treatment (Figure 4E, quantified in Figure 4F) in accord with the reduced levels of ROS and increased MMP following PGZ treatment (Figure 4G,H).

### 3.4. Rescue of Fe-S Cluster Biosynthesis by Pioglitazone Treatment Correlates Well with the Increase in the Enzymatic Activities of Fe-S Protein and with the Improvement of Iron Homeostasis and Inflammation in Sciatic Nerve

**Figure 5:**
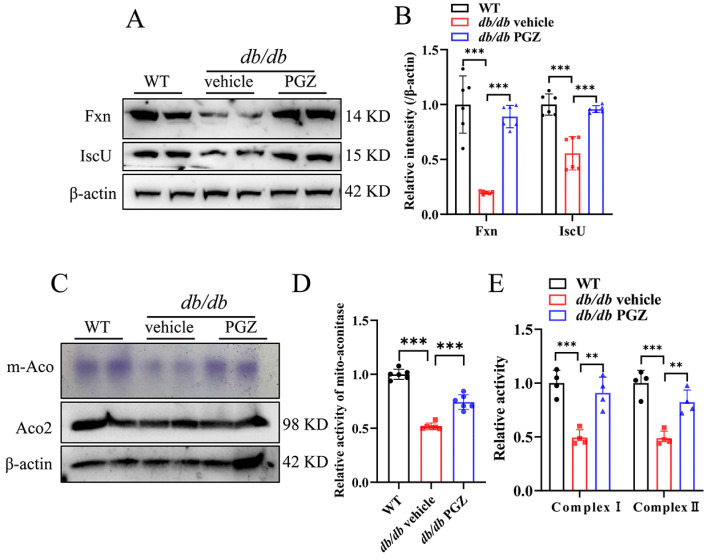
Pioglitazone treatment rescues the capability of biosynthesis of Fe-S clusters and increases the activities of Fe-S-containing enzymes. (**A**) The protein expression levels of Fxn and IscU detected via Western blotting, quantified in (**B**). (**C**) Mitochondrial aconitase activity and corresponding protein levels, quantified in (**D**). (**E**) The activities of mitochondrial complexes I and II. Data were normalized to the WT group (n = 4–6). Values are shown as the mean ± SD. ANOVA was used for significance. ** *p* < 0.01 and *** *p* < 0.001.

To investigate the relevant effects of PGZ on Fe-S biogenesis in vivo, we determined the expression of Fxn and IscU in *db/db* mice after PGZ treatment. *db/db* mice are born with no apparent abnormalities in general appearance or behavior, but they start to develop obesity and glucose intolerance around the age of 4–6 weeks [25]. *db/db* mice at the age of 16 weeks have pathological changes in their sciatic nerve (SCN) [26]. Here, the *db/db* mice at 12 weeks were treated with vehicle or PGZ (25 mg/kg/d, oral administration by gavage) for 4 weeks. Consistent with in vitro experimental results from RSC96 cells with high-glucose treatment, the expression of Fxn and IscU significantly decreased in the SCN of *db/db* mice, and PGZ treatment reversed it well (Figure 5A, quantified in Figure 5B). We then determined whether PGZ improved the activities of mitochondrial Fe-S cluster-containing enzymes and found that both the activities of mitochondrial aconitase and complex I and complex II significantly increased in the SCN of the *db/db* mice (Figure 5C–E).

**Figure 6:**
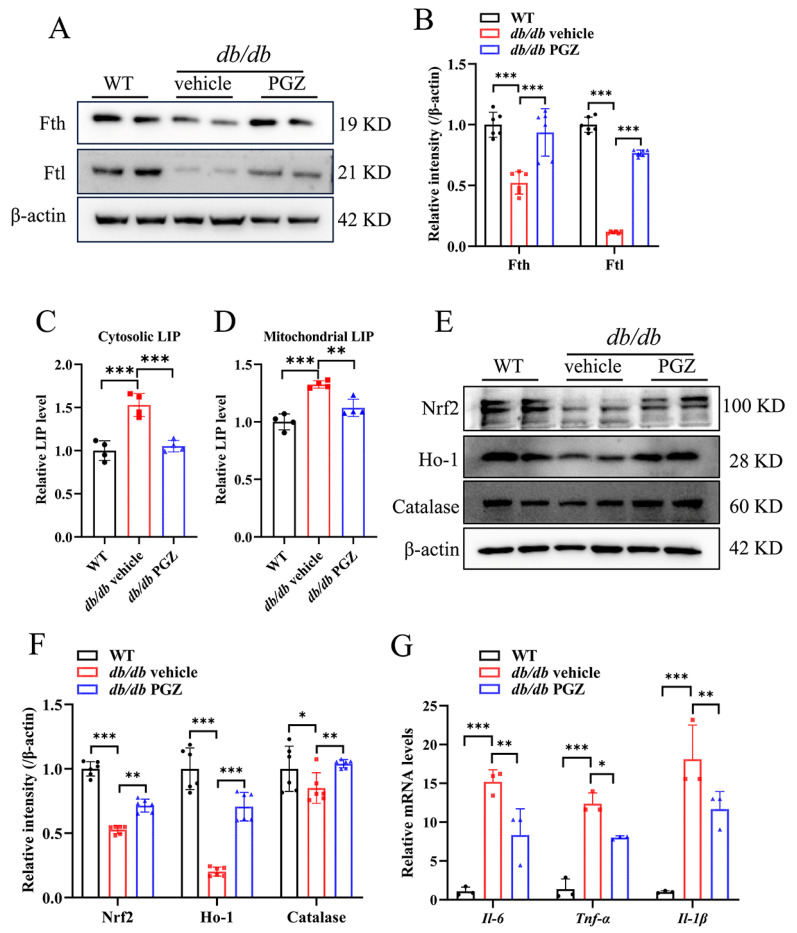
Pioglitazone treatment improves iron homeostasis and alleviates inflammation and oxidative stress in *db/db* mice. (**A**–**E**) *Db/db* mice at 12 weeks were treated with vehicle or PGZ (25 mg/kg/d, oral administration by gavage) for 4 weeks. (**A**) The protein expression of Tfr1, Fpn, Fth, and Ftl detected via Western blotting, quantified in (**B**). (**C**,**D**) The relative levels of cytosolic and mitochondrial LIP. (**E**) The expression levels of oxidative stress-related proteins Nrf2, Ho-1, and catalase detected via Western blotting, quantified in (**F**). (**G**) mRNA expression levels of inflammation-related genes Il-6, Tnf-α and Il-1β. Data were normalized to the WT group. n = 3–6. Values are shown as the mean ± SD. ANOVA was used for significance. * *p* < 0.05, ** *p* < 0.01, and *** *p* < 0.001.

Next, we evaluated the impact of PGZ treatment on iron metabolism and inflammatory levels. Similar to the cell culture results in RSC96 cells, Fth and Ftl expression were significantly rescued after PGZ treatment in the SCN of the *db/db* mice (Figure 6A, quantified in Figure 6B). Accordingly, the relative LIP levels decreased in the cytosol and mitochondria (Figure 6C,D). In addition, the expression of Nrf2 and its target Ho-1 was improved in the SCN, suggesting the enhanced competence of anti-oxidative stress after PGZ treatment (Figure 6E, quantified in Figure 6F). The pathological mechanism of DPN is related to inflammation caused by persistent hyperglycemia [27]. We found that PGZ treatment significantly decreased the mRNA levels of inflammatory cytokines, such as *Il-6*, *Tnf-α*, and *Il-1β*, in the SCN of the *db/db* mice (Figure 6G). These results suggest that PGZ treatment suppressed inflammation and oxidative stress in the SCN of the *db/db* mice.

**Figure 7:**
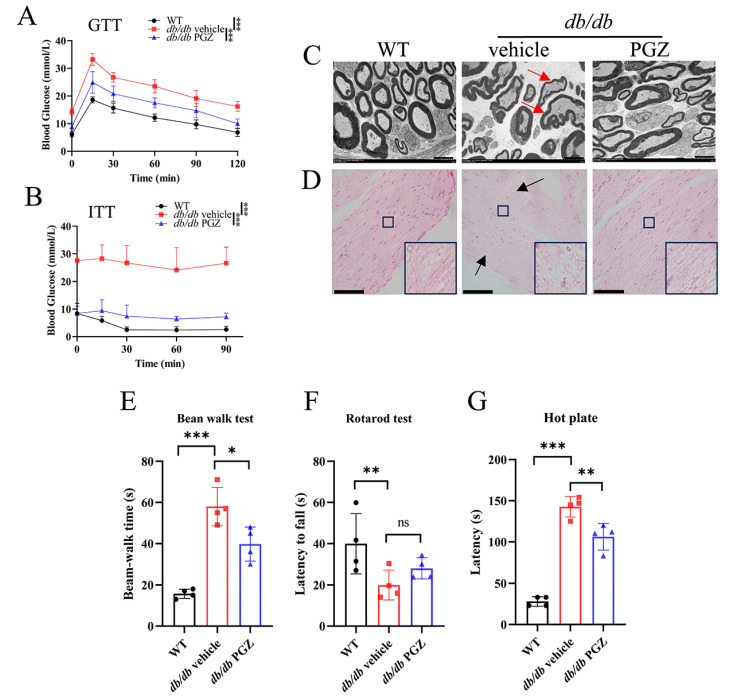
Pioglitazone treatment improves the histological structure of the sciatic nerve and behavior of *db/db* mice. (**A**) The glucose tolerance test (GTT). (**B**) The insulin tolerance test (ITT). (**C**) Representative images of nerve structure in the transmission electron microscopy assays. The thin and deformed myelin sheathes are indicated by red arrows. Scale bar, 5 μm. (**D**) Representative H&E-stained SCN. The rupture sites are indicated by black arrows. Scale bar, 100 μm. (**E**) The beam-walk tests. (**F**) The rotarod tests. (**G**) The hot plate tests. n = 4. Values are shown as the mean ± SD. ANOVA was used for significance. ^ns^ *p* > 0.05, * *p* < 0.05, ** *p* < 0.01, and *** *p* < 0.001.

As expected, glucose intolerance was improved in the PGZ-treated *db/db* mice (Figure 7A and Figure S1A). The insulin tolerance tests (ITT) confirmed that PGZ increased insulin sensitivity in the *db/db* mice (Figure 7B and Figure S1B). Although body weight was significantly higher after PGZ treatment (Figure S1C), the weights of the liver, inguinal white adipose tissue (iWAT), and epididymal white adipose tissue (eWAT) were not significantly affected (Figure S1D–F). However, the postprandial glucose level was reduced considerably, comparable to that of the WT mice (Figure S1G).

Since DPN is usually accompanied by axon degeneration in various nerves, including the SCN [28,29,30], we investigated whether PGZ treatment has a protective effect on diabetic axon degeneration. Transmission electron microscopy was used to observe the ultrastructure of the mouse SCN. Representative cross-section images of axons showed that the myelin sheath was irregular and thinner in the *db/db* mice than in the WT mice and reversed by PGZ treatment, as shown in Figure 7C. The structure of SCN fibers was disordered and damaged, in particular, with rupture/injury in *db/db* mice, and it was significantly improved by PGZ treatment (Figure 7D), revealed through histological H&E staining.

To test behavioral improvement, we examined motor coordination and pain sensitivity. The beam-walk tests showed that *db/db* mice spent more time walking along 22 mm beams. However, PGZ treatment significantly improved this (Figure 7E). During the rotarod tests, the mice exhibited a mild, without significance, prolonged time before falling off post PGZ treatment (Figure 7F). The *db/db* mice showed longer withdrawal latency than the WT mice and significantly shorter withdrawal latency after PGZ treatment (Figure 7G), revealed in the hot plate assays to evaluate pain sensitivity after SCN injury [31]. Altogether, PGZ provides protective effects from the development of DPN, at least partially, via potentiating the Fe-S cluster biogenesis.

## 4. Discussion

An extended period of hyperglycemia-mediated metabolic imbalances, such as oxidative stress, mitochondrial dysfunction, and neuroinflammation, are the main factors associated with the development of DPN [32]. The elucidation of hyperglycemia-related metabolic disruptions remains the foremost target for research. In this study, we found that the expression of Fxn and IscU decreased in the SCN of *db/db* mice (as a DPN mouse model) and high glucose-induced RSC96 cells (as a DPN cell model). Fxn and IscU are the two core components of Fe-S cluster biogenesis machinery. Downregulation of both would severely diminish mitochondrial function, which in Schwann cells is essential for axonal survival and normal peripheral nerve function. Therefore, deficiency of the Fe-S cluster might be, at least, one of the potential mechanisms of human peripheral neuropathies, such as DPN, in this study.

It has been reported that FXN deficiency is a high risk factor in diabetes in FRDA patients and mice [33,34], and DPN is the most common complication of diabetes. However, there have been no reports on a direct connection between the Fe-S cluster and DPN. Here, we found a close correlation in multiple hyperglycemia models. The coupled regulation of Fxn and IscU has been repeatedly observed in FXN-deficient cells, animals, and patients irrespective of whether the deficiency is caused by DNA mutation or HG induced (here and in the work of Li et al. [20]). Both proteins are crucial to maintaining physiological function in the early steps of Fe-S biogenesis through their interaction with the NFS1-ISD11-ACP complex [5]. Downregulation of both Fxn and IscU, therefore, diminished Fe-S cluster biogenesis and Fe-S enzymatic activities in RSC96 cells. This may also be true for *db/db* mice with hyperglycemia, consequently leading to mitochondrial dysfunction, suggesting that Fe-S cluster deficiency may be a potential mechanism causing DPN.

PGZ, a PPARγ agonist, improves a variety of metabolic syndromes related to diabetes and obesity [35]. It enhances insulin sensitivity and effectively reduces plasma glucose levels and glycated hemoglobin (HbA1c). We found that PGZ treatment not only significantly improved glucose metabolism capacity in *db/db* mice but also increased Fxn and IscU expression. We have not seen the PPARγ target nature on the promoters of ISCU and FXN, suggesting indirect regulation. Of note, blockage of iron–sulfur cluster biogenesis by Fxn or IscU depletion in vitro abolished the benefits of PGZ treatment. Thus, animal models, either Fxn- or IscU-deficient mice or *db/db* mice administrated Fxn and/or IscU agonists, need to be utilized for in vivo confirmation. If it is approved, we speculate that PGZ will remarkably improve the function of peripheral nerve tissue in humans, such as SCN, through upregulation of Fe-S cluster biogenesis to enhance mitochondrial function. Long-term diabetes has been linked to distal axonopathy, resulting in compromised sensory nerve function and degenerated peripheral axons in previous reports [29]. If clinical data support our findings, diabetes patients with DPN might consider being prescribed PGZ to reverse the expression of FXN and ISCU to alleviate axon degeneration in addition to the first-line drug metformin. We do not know how many cell types respond to PGZ via an increase in Fxn and IscU expression, but Schwann cells do, and the axonopathy could be rescued, accompanied by significantly reduced inflammation and oxidative stress levels in the SCN of the *db/db* mice. Therefore, DPN patients could benefit from PGZ treatment by lowering blood glucose, increasing FXN and ISCU expression and mitochondrial capacity, and further reducing oxidative stress. FXN protein therapy (CTI-1601, also named Nomlabofusp) is in a phase II clinical trial (https://clinicaltrials.gov, accessed on 1 August 2024) for FRDA patients, who manifest peripheral nerve and central nerve disorders. DPN patients might share a similar physiopathological mechanism to FRDA patients in peripheral nerves. In the case of the efficacy of FXN therapy, DPN patients might take advantage of it. The limitation of this study is that we only used male mice. Whether the results may be expanded to females and to humans needs further investigation.

## 5. Conclusions

In summary, we found reduced expression of Fxn and IscU in DPN cell and animal models. Manipulation of their expression correlates well with DPN symptoms, suggesting that hyperglycemia in patients with diabetes might reduce FXN and ISCU expression to compromise mitochondrial function, contributing to the development of DPN. PGZ or other untested drugs that rescue Fe-S clusters or stabilize Fe-S clusters could have potential benefits for the quality of life of persons with DPN.

## Data Availability

Data are contained within the article and Supplementary Materials.

## Supplementary Materials

The following supporting information can be downloaded at: https://www.mdpi.com/article/10.3390/antiox13091036/s1. Figure S1: PGZ improves glucose metabolism levels in *db/db* mice.
