# Supplementary material for: Downregulation of Iron–Sulfur Cluster Biogenesis May Contribute to Hyperglycemia-Mediated Diabetic Peripheral Neuropathy in Murine Models
Source: Antioxidants (Basel). 2024 Aug 26;13(9):1036. doi: 10.3390/antiox13091036 (PMC11446412; doi:10.3390/antiox13091036)
Supplement: Supplementary file 1 [file antioxidants-13-01036-s001.zip › antioxidants-3097158-supplementary.pdf]

Downregulation of iron-sulfur cluster biogenesis contributes to hyperglycemia-mediated diabetic peripheral neuropathy

Lin Wu[1], Fei Huang[2], Zichen Sun[1], Jinghua Zhang[1], Siyu Xia[2], Hongting Zhao[1], Yutong Liu[3], Yibing Ding[1], Dezhi Bian[2], Kuanyu Li[1,4*], Yu Sun[4*]

[1]Jiangsu Key Laboratory of Molecular Medicine, Medical School, Nanjing University, Nanjing, 210093 P. R. China

[2]Endocrinology Department, Yancheng First People's Hospital, Affiliated Hospital of Medical School, Nanjing University, Yancheng, 224000, P. R. China

[3]State Key Laboratory of Pharmaceutical Biotechnology, Department of Vascular Surgery, Nanjing Drum Tower Affiliated Hospital of Medical School, Nanjing University, Nanjing, 210008, P. R. China

[4]Suqian Scientific Research Institute of Nanjing University Medical School, Nanjing University, Suqian, 223800, P. R. China

Lin Wu and Fei Huang contributed equally to this work.

*To whom correspondence should be addressed:

Jiangsu Key Laboratory of Molecular Medicine, Medical School, Nanjing University, Nanjing 210093, China.

Tel: +86-25-83593192;

Email: likuanyu@nju.edu.cn; sqsunyu@126.com

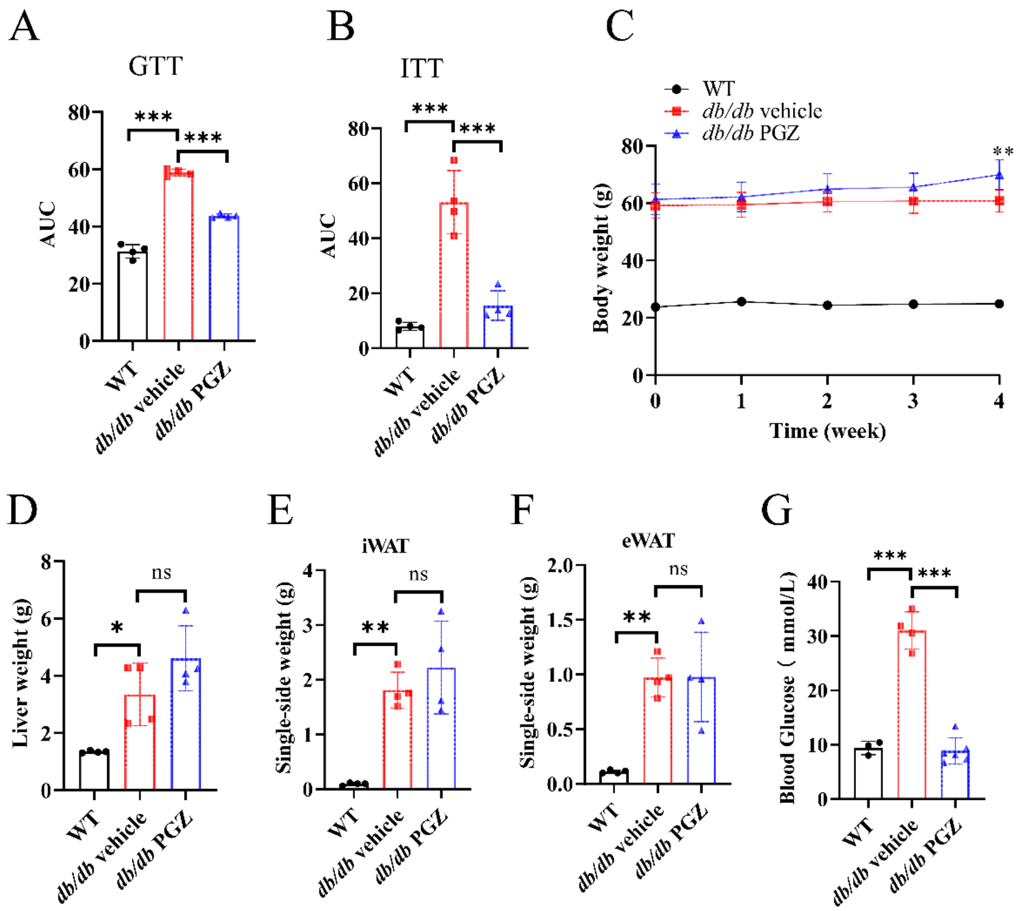

Figure S1. PGZ improves glucose metabolism levels in *db/db* mice.

A-G, *db/db* mice at the age of 12 weeks were treated with vehicle or PGZ (25 mg/kg/d, oral administration by gavage) for 4 weeks (n = 4). A, the area under the curve (AUC) of the GTT. B, the AUC of the insulin tolerance test (ITT). C, the body weight of mice. D, the liver weight. D and F, the single-side weights of iWAT and eWAT. G, postprandial blood glucose of mice. Values are shown as mean ± SD. t-test was used for significance. *P<0.05, **P < 0.01, ***P < 0.001.
